# The Molecular Pathophysiology of Psoriatic Arthritis—The Complex Interplay Between Genetic Predisposition, Epigenetics Factors, and the Microbiome

**DOI:** 10.3389/fmolb.2021.662047

**Published:** 2021-04-01

**Authors:** Ana L. Carvalho, Christian M. Hedrich

**Affiliations:** ^1^Department of Women’s and Children’s Health, Institute of Life Course and Medical Sciences, University of Liverpool, Liverpool, United Kingdom; ^2^Department of Paediatric Rheumatology, Alder Hey Children’s NHS Foundation Trust Hospital, Liverpool, United Kingdom

**Keywords:** psoriasis, psoriatic arthritis, environment, epigenetic, chromatin, nucleosome, inflammation, microbiota

## Abstract

Psoriasis is a symmetric autoimmune/inflammatory disease that primarily affects the skin. In a significant proportion of cases, it is accompanied by arthritis that can affect any joint, the spine, and/or include enthesitis. Psoriasis and psoriatic arthritis are multifactor disorders characterized by aberrant immune responses in genetically susceptible individuals in the presence of additional (environmental) factors, including changes in microbiota and/or epigenetic marks. Epigenetic changes can be heritable or acquired (e.g., through changes in diet/microbiota or as a response to therapeutics) and, together with genetic factors, contribute to disease expression. In psoriasis, epigenetic alterations are mainly related to cell proliferation, cytokine signaling and microbial tolerance. Understanding the complex interplay between heritable and acquired pathomechanistic factors contributing to the development and maintenance of psoriasis is crucial for the identification and validation of diagnostic and predictive biomarkers, and the introduction of individualized effective and tolerable new treatments. This review summarizes the current understanding of immune activation, genetic, and environmental factors that contribute to the pathogenesis of psoriatic arthritis. Particular focus is on the interactions between these factors to propose a multifactorial disease model.

## Introduction

Psoriasis is a systemic autoimmune/inflammatory disorder that primarily affects the skin. It is characterized by effector T cell activation and dysregulated inflammatory cytokine expression (Veale and Fearon, 2018; Surace and Hedrich, 2019). In adulthood, approximately 20% of psoriasis patients develop joint involvement and are diagnosed with psoriatic arthritis (PsA) (Gladman et al., 2005; Rahman and Elder, 2005), an immune-mediated disease affecting the peripheral and axial skeleton. Psoriatic arthritis affects approximately 0.1–3% of the general population (Rahman and Elder, 2005; Ritchlin et al., 2017). In childhood, exact numbers are not known as symptoms of skin psoriasis can be subtle, and psoriatic juvenile idiopathic arthritis (PsJIA), the equivalent to PsA, in approximately 50% of cases manifests with joint disease (sometimes long) before skin symptoms occur (Southwood et al., 1989; Stoll and Nigrovic, 2006).

Classification criteria for PsA (CASPAR) have been developed in 2006, and include confirmed inflammatory articular disease (peripheral joints, spine, or entheses) plus at least three of the following features: current psoriasis (counting double), a history of previous psoriasis or a family history of psoriasis, dactylitis, juxta-articular new bone formation (hands or feet), rheumatoid factor (RF) negativity, and psoriatic nail dystrophy (Taylor et al., 2006; Tillett et al., 2012). A number of comorbidities are associated with psoriasis, including inflammatory bowel disease (IBD), obesity, metabolic syndrome, cardiovascular disease, and depression (Perez-Chada and Merola, 2020). Both skin psoriasis and PsA can significantly affect patients’ quality of life, are associated with increased morbidity and mortality, and represent a considerable economic burden to healthcare systems (Ritchlin et al., 2017).

Although psoriasis and PsA can be seen as presentations along a clinical spectrum of “psoriatic disease,” severity of skin disease only modestly correlates with joint activity (Mease et al., 2019). Although incompletely understood, psoriasis and PsA are multifactor disorders with genetic and epigenetic contributors, and environmental triggers that are associated with the initiation and propagation of inflammation (Cafaro and Mcinnes, 2018; Talotta et al., 2019). Understanding individual pathomechanistic factors and their interplay is essential for the identification and validation of disease- and outcome-specific biomarkers, and the introduction of new individualized, target-directed, effective, and tolerable treatments.

In the following, we will discuss the current understanding of genetic, epigenetic and environmental factors in psoriasis and PsA, including the complex involvement of the microbiome. Special focus is on PsA, as it may represent a particularly severe form of psoriatic disease with a more pronounced clinical and molecular phenotype.

## Immune (DYS-)Regulation

Immune dysregulation, altered cytokine expression, and cellular phenotypes are key features of psoriasis and PsA (Belasco et al., 2015; Blauvelt and Chiricozzi, 2018).

### Dysregulated Cytokine Expression

In PsA, immune activation and associated pro-inflammatory cytokine expression are most pronounced at the level of joint/synovium and skin (Belasco et al., 2015), where increased expression of tumor necrosis factor (TNF)-α can be seen. Expression of the effector cytokine interleukin-17 (IL-17) is more pronounced in the skin, while pro-inflammatory IL-6 expression is higher in the synovium. Notably, cytokine expression in the synovium of PsA patients is more closely related to gene expression patterns in affected skin as compared to gene expression in the synovium of patients with other forms of chronic autoimmune arthritis, including rheumatoid arthritis (RA) and osteoarthritis (OA) (Belasco et al., 2015), suggesting cytokine expression to be somewhat disease rather than symptom (arthritis) specific.

**FIGURE 1:**
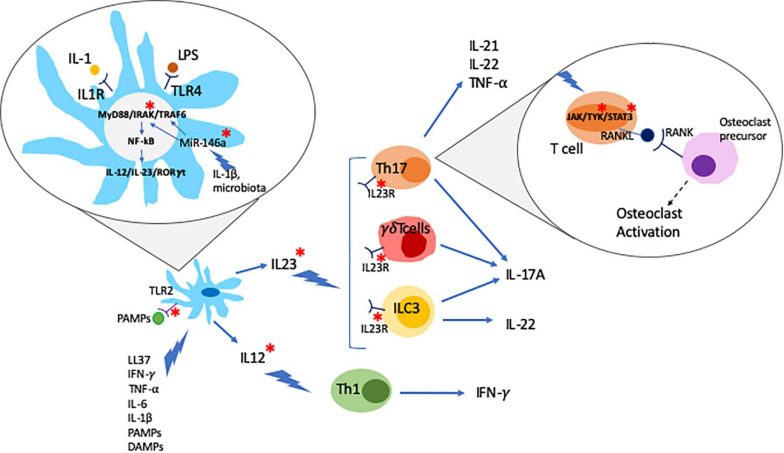
Psoriatic arthritis is a multifactor disease. Dendritic cells (DC) play a central role in the pathogenesis of psoriatic arthritis. DC activated by different stimuli produce and secrete IL-23 and IL-12 which further stimulate Th17, γδT cells and ILC3 and Th1 cells, respectively, leading to the production of inflammatory cytokines. Inflammatory cytokines can act by activating the JAK/STAT signaling pathway which regulates the expression of RANKL. RANKL binds to RANK activating osteoclast precursors. Environmental triggers such as changes in the microbiota, PAMPs (pathogen associated molecular patterns), and LPS can trigger the development of inflammatory cascades such as the MyD88/IRAK/TRAF6 pathway. Epigenetic mechanism, namely non-coding RNAs (e.g., MiR-146a), has been suggested as a critical link between environmental triggers and aberrant inflammatory responses. PsA genetic associations are highlighted with a red *.

The IL-23/T helper (Th)17 axis is widely accepted to play a key role in the pathogenesis of psoriasis, particularly skin disease. IL-23 is a heterodimeric cytokine composed of the p19 and p40 subunits that are mainly secreted by innate immune cells, such as dendritic cells (DCs) and macrophages (Khader and Thirunavukkarasu, 2019). The IL-23R receptor is also composed of two subunits, IL-23R and IL-12Rb1, and expressed on the surface lymphoid cells, including Th17 cells, and a subset of myeloid cells, including macrophages and DCs (Awasthi et al., 2009). IL-23R activation induces phosphorylation of protein kinases Jak2 and Tyk2, which mediate the activation of the transcription factors STAT3 and RORγ, both promoting the differentiation of IL-17 producing Th17 cells (Ivanov et al., 2006; Liang et al., 2014). The use of the small molecule inhibitor tofacitinib (approved for the treatment of PsA, see below) inhibits the production of IL-17 and interferon-γ (IFN-γ) in peripheral blood and synovial CD4^+^ T cells from PsA patients in a dose-dependent manner (Maeshima et al., 2012). Notably, increased pathological activation of Th17 cells has been linked with inflammation and tissue damage in psoriasis and other autoimmune/inflammatory conditions, such as SLE (Talaat et al., 2015; Zhou et al., 2018). The effector cytokine IL-17A can be produced as IL-17A/A homodimer or as a heterodimer together with its homolog IL-17F (IL-17A/F). Of note, IL-17A/A is believed to be of higher pro-inflammatory capacity as compared to IL-17A/F, and in some conditions (namely systemic lupus erythematosus, SLE) the balance is shifted toward IL-17A/A. IL-17A/A and IL-17A/F are secreted by effector T cells, including CD4^+^ Th17 cells, innate lymphoid cells (ILCs), and neutrophils, mainly in response to stimulation with IL-23 (Wright et al., 2007; Cua and Tato, 2010; Hedrich et al., 2012; Pantelyushin et al., 2012). IL-17 binds to and activates the IL-17 receptor (IL-17R) stimulating lymphocytes, monocytes, fibroblasts, and keratinocytes, thereby promoting inflammation and tissue damage (Miossec and Kolls, 2012; Blauvelt and Chiricozzi, 2018). In particular, IL-17 together with TNF-α, induce the expression of matrix metalloproteinases (MMPs) in synovial fluid and cartilage, which mediates loss of collagen structures and cartilage surface erosion (Koenders et al., 2011). IL-17 can also stimulate receptor activator of nuclear factor kappa-B ligand (RANKL) expression in osteoblasts and its ligation to the receptor activator of nuclear factor kappa-B (RANK) in osteoclast precursors promoting osteoclast differentiation and activation (Kotake et al., 2012) (Figure 1).

While the exact reasons for pathological immune cell activation and cytokine expression are not known, polymorphisms in associated genes and epigenetic alterations are suspected to be involved and will be discussed in sections Genetic Factors and Epigenetic.

### Innate Immune Cell Dysregulation

The development and maintenance of psoriatic disease involves the activation and close interaction of innate and adaptive immune cells (Hedrich, 2016). Macrophages are part of the first line of defense against pathogens, and play a central role in innate immune responses. The synovial environment contains various macrophage subpopulations. In RA, macrophages are associated with both joint homeostasis and chronic inflammation (Kurowska-Stolarska and Alivernini, 2017). In PsA, the different populations of macrophages and respective roles in the different disease stages of disease haven’t been finally characterized. However, recent work suggests that macrophages are important mediators of inflammation in PsA and may be key in the cross-talk between tissues affected and the skin-to-joint transition of inflammation (Van Raemdonck et al., 2020). In murine models, the micro-RNA Let7b, a toll-like receptor-7 (TLR-7) ligand, induces skin inflammation that is characterized by infiltration with CD68^+^ macrophages and increased the expression of pro-inflammatory cytokines IL-1β, IL-6, and IL-12 in joints. Stimulated skin macrophages are characterized by a shift in their metabolism toward glycolysis. Treatment with specific glycolytic inhibitors disrupted the TLR7-driven inflammatory phenotype and decreased pro-inflammatory cytokine production (Van Raemdonck et al., 2020).

Natural killer (NK) cells are part of the immune response against tumors and virus infected cells. In PsA, NK cell numbers are reduced in the peripheral blood (Conigliaro et al., 2014) suggesting their recruitment to the synovium. In the synovium of PsA patients, NK cells produce granulocyte macrophage colony-stimulating factor (GM-CSF) that mediates the differentiation of monocytes into DCs, key links between the innate and adaptive immune system (Zhang et al., 2007). NK cells can be divided into two main subtypes, CD56^*bright*^ and CD56^*dim*^ NK cells. CD56^*dim*^ NK cells are mainly circulating cells that are characterized by reduced cytotoxicity and increased production of cytokines. CD56^*bright*^ NK cells express CCR7 and CD62L homing receptors that guide cells to infiltrate tissues, such as lymph nodes or the inflamed synovium (Bjorkstrom et al., 2016). Indeed, CD56^*bright*^ NK cells expressing CD69 and NKp44 are present in inflamed joints in PsA (De Matos et al., 2007) where they contribute to bone destruction, the induction of effector T cell responses, and fibroblast proliferation (Soderstrom et al., 2010). Interestingly, CD69 and NKp44 expression is stimulated by microbial peptidoglycan ligation to NK cells through TLR-2 (Esin et al., 2013). The involvement of NK cells in the pathophysiology of PsA is further underscored by the presence of PsA specific genetic variants in *KIR2DL2* and *KIR2DS2* (Chandran et al., 2014). The *KIR2D* gene encodes for the cell immunoglobulin-like receptor KIR2D that is mainly expressed on NK cells (also see Genetic Factors section). KIRs specifically ligate HLA-C epitopes, thereby determining NK cell activity against cells that miss the target HLA ligands (Ichise et al., 2017).

Gamma delta T cells (γδT cells) are a small subset of peripheral T cells present in epithelia and mucosal tissues, where they are involved in the defense against pathogens (Nielsen et al., 2017). IL-17 producing γδT cells express chemokine receptors CCR6 and CCR2, which, in PsA and other inflammatory conditions, can direct them to the skin and/or synovium (McKenzie et al., 2017). γδT cells contribute to inflammation and tissue damage in autoimmune/inflammatory diseases, including psoriasis and colitis (Nguyen et al., 2019), as they are potent producers of IL-17, mainly upon IL-23 stimulation (Ono et al., 2016). PsA patients exhibit increased numbers of γδT cells in circulation and inflamed joints, where they present a unique transcription factor profile, including increased RAR-related orphan receptor (ROR)γt expression. RORγt is a master regulator transcription factor of Th17-differentiation and activation, responsible for inducing the transcription of IL-17 in CD4^+^ T cells. The selective inhibition of RORγt with the small molecule BIX119 suppresses IL-17 production in Th17, iNKT, and γδT cells (Venken et al., 2019). In line with these observations, RORγt-deficient mice lack Th17 cell tissue infiltration and exhibit reduced inflammation and damage (Ivanov et al., 2006).

Innate lymphoid cells (ILCs) are a recently identified and rare immune cell subset that is characterized by the absence of recombination activating gene expression (Geremia and Arancibia-Carcamo, 2017). ILCs recruit to epithelial surfaces and respond to stress signals, including bacterial and viral infection, thus playing a critical role in the maintenance of epithelial homeostasis. ILCs comprise three sub-lineages with distinct distributions across mucosal sites and patterns of cytokine production. PsA patients exhibit increased numbers of circulating total ILCs, ILC1, ILC2, and ILC3 subpopulations when compared with healthy controls (Soare et al., 2018). ILC1 are a tissue-resident subset that plays a crucial role in viral infection response by mainly producing IFN-γ. ILC2 predominantly synthetize immune-regulatory IL-4, IL-5, IL-9, and IL-13. Their role in the resolution phase of inflammation has been described in murine inflammation models and requires investigation in PsA (Diefenbach et al., 2014; Rauber et al., 2017). ILC3 are primarily found in the intestinal mucosa; they are stimulated by IL-23 and produce IL-17 and IL-22 (Diefenbach et al., 2014). Of note, NKp44 expressing ILC3 are increased in the peripheral blood (as compared with healthy controls) and the synovial fluid (as compared to RA patients) of PsA patients (Leijten et al., 2015; Soare et al., 2018). Lastly, a reduced peripheral blood ILC2/ILC3 ratio correlates with the clinical disease activity scores in PsA patients, further suggesting a key role of ILCs and their balanced cytokine expression (Leijten et al., 2015; Soare et al., 2018).

### Effector T Cells

Effector T helper cell populations have been implicated in the pathophysiology and tissue damage in psoriasis and PsA.

In PsA, effector CD4^+^ Th1 cells produce IFN-γ, a pro-inflammatory cytokine that activates antigen-presenting cells (APCs). In turn, APCs contribute to Th17 cell differentiation and the induction of IL-17 inflammatory cascades, thereby promoting skin and bone pathology (Kelchtermans et al., 2009). Notably, also CD3^+^ TCR^+^ CD4^–^ CD8^–^ (“double negative”) T cells that can arise from CD8^+^ T cells are infiltrating the skin of psoriasis patients and produce the effector cytokine IFN-γ, likely also contributing to aforementioned inflammatory cascades (Brandt et al., 2017). However, their involvement in joint inflammation has not been investigated yet.

Effector Th17 lymphocytes are increased in the peripheral blood and synovial fluid of patients with PsA (Wei et al., 2007; Jandus et al., 2008; Volpe et al., 2008). Furthermore, synoviocytes of PsA patients show increased expression of IL-17 receptor (IL17R) as compared to cells from osteoarthritis patients (Raychaudhuri et al., 2012), underscoring the role of IL-17 in the inflammatory process and damage accrual in PsA.

Recently appreciated Th9 cells are one of the main producers of IL-9, an effector cytokine involved in hematopoietic cell proliferation and the regulation of apoptosis (Kaplan et al., 2015). The expression of IL-9 is significantly upregulated in gut biopsies from PsA patients when compared to those from ankylosing spondylitis (AS) and Crohn’s disease (CD). In the gut, IL-9 gut stimulates the proliferation of Paneth and epithelial cells (and overexpression of IL-23p19) (Ciccia et al., 2016b). Furthermore, IL-9 and IL-9 producing CD3^+^ T cells are increased in the synovial fluid of PsA when compared to osteoarthritis patients. This is of particular relevance, as IL-9 induces proliferation of peripheral blood and synovial CD3^+^ T cells from PsA patients through activation of the PI3K/Akt/mTOR signaling pathway (Kundu-Raychaudhuri et al., 2016). Confirming aforementioned observations, Ciccia et al., identified an IL-9 and IL-9R gene expression signature in the synovial tissue of PsA patients (Ciccia et al., 2016b). Intriguingly, IL-9 is also involved in restoring the immune homeostasis during the resolution stage of arthritis (Rauber et al., 2017). Thus, it will be of key importance to fully understand the role of IL-9 in the molecular and cellular pathways at different stages of psoriasis and PsA.

Lastly, mucosa-associated invariant T cells (MAIT) are defined by CD161^*hi*^ and CD26^*hi*^ surface expression. Notably, while predominantly being CD4^+^ in RA, PsA patients exhibit CD8^+^ MAIT in the synovial fluid that express IL23R and proliferate in response to stimulation with IL-23. Thus, CD8^+^ MAIT may contribute to inflammation and tissue damage in PsA through the production of IL-17 (Raychaudhuri et al., 2020).

### Interconnections Between Innate and Adaptive Immune Responses

Dendritic cells are a critical link between the innate and adaptive immune system. They have been associated with various autoimmune/inflammatory diseases, including psoriasis, PsA, SLE, and others (Jongbloed et al., 2006; Hedrich, 2016; Xiao et al., 2020). In T cell-mediated inflammatory disease, DCs play a crucial role in activating effector T cells (Hedrich, 2016). Depending on their individual progenitor cells, DCs can be divided in myeloid dendritic cells (mDC) or plasmacytoid dendritic cells (pDC). mDCs regulate pro-inflammatory responses through the induction of T helper and cytotoxic T lymphocyte responses against bacterial and viral infection. pDCs typically produce type I interferons (type I IFN) that plays an important role in anti-viral responses. In the peripheral blood of PsA patients, the number of pDCs is reduced when compared to healthy controls. Conversely, pDCs and mature mDCs, expressing DC80, CD83, and CD86, are abundant in the synovial fluid of PsA patients suggesting their recruitment to sites of inflammation (Jongbloed et al., 2006). Moreover, the ratio between mDCs and pDCs is increased in the synovial fluid of PsA patients, which is in agreement with increased expression of IFN-γ in this compartment. In turn, IFN-γ stimulates the differentiation of mDCs (Jongbloed et al., 2006; Lande et al., 2007). Interestingly, synovial fluid DCs in PsA overexpress TLR-2 which is associated with activation of the nuclear factor kappa B (NFκB) pathway. The relevance of this signaling cascade is highlighted by the association of missense mutations in *TLR2* (rs5743708) and *CARD14* genes with psoriasis (Jordan et al., 2012; Oliveira-Tore et al., 2019). Caspase recruitment domain-containing protein 14 (CARD14) is a regulator of NFκB signaling (also see Genetic Factors section).

In PsA, DCs can be stimulated by the innate antibacterial protein LL37 that is produced by infiltrating neutrophils. In this context, DC activation is mediated through TLR7/8/9, resulting in the secretion of type I IFNs (Lande et al., 2007; Frasca et al., 2018). Furthermore, DCs can be stimulated by TNF-α, INF-γ IL-6 and IL-1β, pathogen-associated molecular patterns (PAMPs), and damage-associated molecular patterns (DAMPs) to produce pro-inflammatory IL-23 and IL-12. IL-12 promotes effector Th1 lymphocyte differentiation that produce IFN-γ, which in turn enhances cytokine production in DCs. IL-23 favors Th17 cells and the synthesis of IL-17A, IL-17F, IL-21, and IL-22 (Liang et al., 2006; Pene et al., 2008) (Figure 1).

## Genetic Factors

Mutations in single genes that result in psoriasis are rare and only affect a small subset of psoriasis patients. Monogenic disease can be caused by gain of function mutations in caspase recruitment domain-containing protein 14 (*CARD14*) located in the *PSORS2* region on chromosome 17q25 (Jordan et al., 2012; Signa et al., 2019). Homozygous mutations in *LPIN2* result in the autoinflammatory disease Majeed syndrome, characterized by chronic recurrent multifocal osteomyelitis, skin inflammation, and dyserythropoietic anemia (Ferguson et al., 2005). Interestingly, mono-allelic *LPIN2* variants in family members of Majeed syndrome patients associate with an increased risk for psoriasis (Ferguson et al., 2005).

Most psoriasis patients have a genetic predisposition that increases the risk for developing psoriasis, but are by themselves not strong enough to confer disease (O’Rielly et al., 2019) (Table 1). The best characterized genetic associations predisposing to psoriasis are relevant to both skin psoriasis and PsA, and account for approximately one-third of the genetic contribution, and affect the Human Leukocyte Antigen (HLA) cluster. Although the major histocompatibility complex has been associated with susceptibility to both psoriasis and PsA, frequencies of HLA-B and HLA-C alleles differ between skin psoriasis and musculoskeletal disease (Winchester and FitzGerald, 2020). In a cohort of 359 PsA and 212 skin psoriasis patients, HLA-C^∗^06 associated with disease limited to the skin, while HLA-B^∗^39, HLA-B^∗^38, and HLA-B^∗^08 associated with PsA (Winchester et al., 2012). Association of HLA-C^∗^06 with skin psoriasis was confirmed in a study involving 2699 psoriasis patients and 2107 controls (Das et al., 2015). Another study involving 712 PsA, 335 psoriasis patients and 713 healthy controls found HLA-B^∗^27 and HLA-B^∗^38 frequencies to be significantly higher in patients with PsA as compared to both psoriasis patients and healthy individuals (Eder et al., 2012). The role of HLA-B^∗^27 in the expression of PsA has been confirmed by various authors (Alenius et al., 2002; Elkayam et al., 2004; Liao et al., 2008; Minh et al., 2019). Lastly, different HLA susceptibility genes have been associated with specific clinical features. Axial involvement in PsA has been associated with HLA-B^∗^27, HLA-B^∗^39, HLA-B^∗^38, and HLA-B^∗^08 (Queiro et al., 2006; Eder et al., 2012), while enthesitis was associated with HLA-B^∗^27, and dactylitis with HLA-B^∗^27 and HLA-B^∗^08-HLA-C^∗^07 (Haroon et al., 2016). As underscored by their presence in a meaningful subset of healthy individuals, HLA-B^∗^27 average frequency in the worldwide population is 8% although this varies with demographic distribution, HLA variants increase the risk for autoimmune/inflammatory disease, but are not exclusively causative (Akassou and Bakri, 2018). Indeed, most of the currently known genetic associations in skin psoriasis (extending beyond the HLA cluster) only have relatively small contribution to disease expression. Nonetheless, their study is necessary to understand immune pathways and physiological/metabolic processes involved in the development and maintenance of disease, and to allow future prediction of disease courses (Table 1) (Cafaro and Mcinnes, 2018; Veale and Fearon, 2018; O’Rielly et al., 2019). For example, a single-nucleotide polymorphism (SNP) in the collagen10A1 gene (rs3812111c.155A > T, *COL10A1*) has been associated with PsA. *COL10A1* is a type X collagen gene that is expressed in chondrocytes, highlighting the involvement of cartilage/bone metabolism in PsA (Caputo et al., 2020a).

**TABLE 1:** Genetic associations in PsA.

Gene	Description	Pathway	Disease association			References
			PsA	Ps	Others	
*IL12B*	Interleukin 12B (IL-12β)	IL-23/Th17	x	x	IBD, RA, SLE, Behcet’s disease	Bowes et al., 2015
*IL23R*	Interleukin 23 receptor (IL-23R)	IL-23/Th17	x	x	IBD, RA, SLE, BD, MS	Bowes et al., 2015
*IL23A*	Interleukin 23, alpha subunit p19 (IL-23A)	IL-23/Th17	x	x	IBD, SLE, MS	Bowes et al., 2015
*STAT3*	Signal transducer and activator of transcription 3	IL-23/Th17	x	x	IBD, SLE, MS	Cenit et al., 2013
*TYK2*	Tyrosine kinase 2	IL-23/Th17	x	x	SLE, MS, IBD, RA	Bowes et al., 2015
*TRAF3IP2*	TRAF3 interacting protein 2		x	x	RA, SLE	Bowes et al., 2015
*IL2/IL21*	Interleukin 2	IL-21 pathway	x	x	RA, MS	
*TNIP1*	TNFAIP3 interacting protein 1	TNF-induced NFkB-dependent gene expression	x	x	SLE, BD,	Bowes et al., 2015
*REL*	V-rel avian reticuloendotheliosis viral oncogene homolog	Part of NFkB complex	x	x	BD	Ellinghaus et al., 2012
*FBXL19*	F-box and leucine-rich repeat protein 19	Inhibits NFkB signaling	x	x		Stuart et al., 2010
*IRAK*	Interleukin-1 receptor-associated kinase 1	T cell activation and cytokine signaling	x		RA, SLE,	Chatzikyriakidou et al., 2010
*KIR2DL2*	Killer cell immunoglobulin-like receptor, two domains, long cytoplasmic tail, 2	NK cell activity	x			Chandran et al., 2014
*KIR2DS2*	Killer cell immunoglobulin-like receptor, two domains, short cytoplasmic tail, 2	NK cell activity	x		RA	Chandran et al., 2014
*TLR2*	Toll-like receptor 2	Microbial tolerance	x		SLE, IBD	Oliveira-Tore et al., 2019
*MICA*	MHC class I polypeptide-related sequence A	T cell activation and cytokine signaling	x	x	RA, IBD, SLE	Queiro et al., 2014
*MICB*	MHC class I polypeptide-related sequence B	T cell activation and cytokine signaling	x		RA, IBD, SLE	Queiro et al., 2014
HLA-B*08	Human Leukocyte Antigen B08	T cell activation and cytokine signaling	x			Winchester et al., 2012
HLA-B*27	Human Leukocyte Antigen B27	T cell activation and cytokine signaling	x	x		Alenius et al., 2002; Elkayam et al., 2004; Liao et al., 2008; Eder et al., 2012; Minh et al., 2019
HLA-B*38	Human Leukocyte Antigen B38	T cell activation and cytokine signaling	x			Eder et al., 2012; Winchester et al., 2012
HLA-B*39	Human Leukocyte Antigen B39	T cell activation and cytokine signaling	x			Winchester et al., 2012
*COL10A1*	Collagen10A1	Cartilage/bone metabolism	x			Caputo et al., 2020b
*mRNA-146a*	MiRNA-146a	Microbial tolerance	x			Maharaj et al., 2018
*COL6A5*	Collagen6A5	Angiogenesis and vascular proliferation	x			Caputo et al., 2020b
*COL8A1*	Collagen8A1	Angiogenesis and vascular proliferation	x			Caputo et al., 2020b

*PsA, psoriatic arthritis; Ps, psoriasis; others, other autoimmune/inflammatory diseases associated with genetic variants of these genes; IBD, inflammatory bowel disease; RA, rheumatoid arthritis; SLE, systemic lupus erythematosus; BD, Behcet disease; MS, multiple sclerosis.*

Most genetic variants associated with skin disease are affecting immune inflammatory responses, differentiation of structural components of tissues such as angiogenesis, bone metabolism, and microbial tolerance (Cafaro and Mcinnes, 2018; Veale and Fearon, 2018; O’Rielly et al., 2019). Of note, several psoriasis and PsA-associated gene variants, including variants affecting the *IL12B* (5q31.1eq33.1), *IL23A* (12q13.3), *TYK2*, *STAT3*, and *TRAF3IP2* genes, enhance T cell activation and cytokine signaling pathways mostly related with the IL-23/-17 axis (Bowes et al., 2015; Bojko et al., 2018). Additional polymorphisms in *JAK2*, *SOCS1*, and *ETS1* are exclusively associated with psoriasis (Cafaro and Mcinnes, 2018). Polymorphisms in *IL23R* have been linked to genetic susceptibility in a number of human autoimmune diseases including PsA, psoriasis, and IBD (Table 1) (Huffmeier et al., 2009; Kim et al., 2011).

As mentioned above, the majority of genetic variants associated with psoriasis or PsA are not strong enough to explain the development of disease. Thus, additional factors are required to trigger disease expression in a genetically predisposed individual. Environmental factors have been described to contribute to the pathophysiology of psoriatic disease and “blamed” for inter-individual variability (Roszkiewicz et al., 2020). However, the exact molecular mechanisms through which the environmental factors trigger disease in a genetic susceptible individual are not completely clear.

Genetic associations in psoriasis and PsA have been long recognized. However, factors determining whether genetically susceptible individuals develop disease remain largely unknown. Ongoing and future research (will) address the question of how relatively common genetic variants, in the context of environmental or additional host factors, contribute to the expression of clinical disease, while other individuals carrying the same or closely related variants remain healthy or develop other inflammatory conditions.

## Epigenetic Alterations

Epigenetic mechanisms are heritable, but reversible changes affecting gene expression without altering the underlying DNA sequence. They include DNA methylation, post-translational histone modifications and non-coding RNAs (Goldberg et al., 2007; Hedrich and Tsokos, 2011; Surace and Hedrich, 2019). The study of epigenetics has added another layer of complexity to understanding the physiopathogenesis of various immune-mediated disorders, including psoriasis and PsA (Table 2). While many questions still remain unanswered, the study of disease-associated epigenetic changes has elucidated heritable and acquired events not explained by genetics (alone). Furthermore, epigenetic mechanisms have been identified as “missing link” between trigger events, such as changes to the microbiome, and resulting aberrant immune responses (Chen et al., 2017).

**TABLE 2:** Reported epigenetic events in PsA patients.

Epigenetic event	Compartment/tissue	N patients	N controls	Findings	References
DNA methylation	PBMC	3 PsA taking Methotrexate	1 PsA taking other drugs	Inflammation in PsA is characterized by a global hypomethylation that is reversed by treatment with Methotrexate.	Kim et al., 1996
DNA methylation	Whole blood	24	24	Genes MICA, IRIF1, PSORS1C3, and TNFSF4 are hypermethylated and PSORS1C1 is hypomethylated in paternally compared to maternally transmitted disease.	O’Rielly et al., 2014
DNA methylation	Sperm	23 Ps, 13PsA	18 HC	754 differentially methylated regions (DMRs) in PsA vs. HC controls, and 86 between PsA and Ps. DMRs associated with skin and/or joint disease (MBP, OSBPL5, SNORD115, HCG26), and joint disease (IL22, ELF5, PPP2R2D, PTPRN2, HCG26)	Pollock et al., 2019
Histone modifications	Serum	23 PsA 1 year after anti-TNF-α treatment	Same 23 PsA before starting Anti-TNF-α treatment	*de novo* ANA reactivity in 4 patients (after starting anti-TNF-α) and anti-chromatin in other 3 patients.	Viana et al., 2010
Histone modifications	Blood	14 AS, 6 PsA	6 HC	Targeting bromodomains in histones (HDCAC inhibitor trichostatin A (TSA) and JQ1 (a novel panBET bromodomain HAT inhibitor): CBP30 reduces production of IL17A, GM-CSF and IL10 in both PsA and healthy controls.	Hammitzsch et al., 2015
Histone modifications	PBMC	11 PsA	42 HC	Global histone acetylation of H4 but not H3 is decreased in PsA comparing with HC. H3K4 methylation was significantly increased in PsA comparing with controls.	Ovejero-Benito et al., 2018
MiRNA (miRNA-146a)	PBMC	29 PsA	66 HC	Study of 2 polymorphisms in IRAK1, which is a miRNA-146a target. Significant differences were found in IRAK1 rs3027898 polymorphism distribution between patients with PsA when compared with HC. And higher, but not significant difference in IRAK1 rs1059703 genotypes of PsA comparing with HC.	Chatzikyriakidou et al., 2010
MiRNA (miRNA-21-5p)	PBMC	PsA	ERA and HC	In early PsA, a 19- (vs. HC) and 48- (vs. ERA) miRNA signature was identified. MiR-21-5p was found up-regulated both in early PsA and ERA.	Ciancio et al., 2017
MiRNA (miRNA-126-3p)	PBMC	23 PsA	15 HC and 7 RA	miRNA-126-3p is downregulated in PsA active patients and its overexpression induces a decrease expression in PsA associated genes.	Pelosi et al., 2018
MiRNA (miRNA-146a)	Whole blood	116 PsA	100 HC	The miRNA-146a rs2910164 variant C-allele frequency in PsA patients was significantly higher vs. healthy controls.	Maharaj et al., 2018
MiRNA	Exosomes isolated from plasma	30 PsA, 15 Ps, 15 RA, 15 GA	15 HC	82 microRNAs derived from plasma exosome were found specifically in PsA patients, and 36 in common with Ps, RA and GA.	Chen et al., 2019
MiRNA (miR-23a-27a-24-2)	Synovial fluid and PBMC	PsA	OA	Differential expression of the miR-23a-27a-24-2 cluster in PsA synovial tissue and PBMCs compared to osteoarthritis and correlated with disease activity.	Wade et al., 2019a
MiRNA (miRNA-146a-5p)	PBMC isolate monocytes	34 PsA,	17 Ps and 34 HC	MiRNA-146a-5p expression in CD14^+^ monocytes derived from PsA patients correlates with clinical efficacy (biologic treatment), and induction of osteoclast activation and bone resorption.	Lin et al., 2019
MiRNA	Serum	20 PsA 31 PsA for drug response evaluated at 0 and 3, 6 or 9 months	20 HC	Six miRNA (miRNA-221-3p, miRNA-130a-3p, miRNA-146a-5p, miRNA-151-5p, miRNA-26a-5p and miRNA-21-5p) are higher in PsA compared to healthy controls. Higher baseline levels of miRNA- 221-3p, miRNA-130a-3p, miRNA-146a-5p, miRNA-151-5p and miRNA-26a-5p were associated with therapeutic response.	Wade et al., 2020
MiRNA (miRNA-30e-5p)	Plasma EVs	14 PsA	15 Ps	Significantly lower levels of plasma EV miRNA-30e-5p in PsA vs Ps.	Pasquali et al., 2020
MiRNA (miRNA-941)	PBMC isolate monocytes	40 PsA	40 Ps and 40 HC	The miRNA-941 is upregulated in CD14^+^ monocytes from PsA patients.	Lin et al., 2020
MiRNA (miRNA-29 and miRNA-Let7B)	Synovial fluid	PsA	OA	MiRNA-29 and miRNA-Let7B are increased in PsA synovium fluid. MiRNA-Let7b promotes skin inflammation and joint inflammation through Th1 cells and CD68^+^ M1 macrophages amplification process.	Van Raemdonck et al., 2020

*PubMed search for: psoriatic/psoriasis arthritis + epigenetics/DNA methylation/histones/miRNA. Ps, psoriasis; PsA, psoriatic arthritis; AS, ankylosing spondylitis; ERA, enthesitis-related arthritis; OA, osteoarthritis; HC, healthy controls; GA, gouty arthritis.*

Currently, studies investigating DNA methylation and/or histone modifications in PsA are scarce. However, some studies have addressed relevant questions, such as the role of epigenetics on drug responders/non-responders and on differences in paternal vs maternal transmission of PsA (Table 2) (Kim et al., 1996; O’Rielly et al., 2019). Non-coding RNAs are regulatory RNA molecules that have gained great attention for their proposed roles in the regulation of transcription and cell-to-cell communication (Saliminejad et al., 2019). Non-coding RNA signatures in PsA have mostly been linked with microbial tolerance, T cell activation and cytokine signaling, cell proliferation and inflammation, and cartilage/bone metabolism (Lin et al., 2019; Van Raemdonck et al., 2020; Wade et al., 2020).

### DNA Methylation Patterns in PsA

DNA methylation is mediated by the covalent ligation of a methyl group to the 5′carbon position of cytosine in cytosine-phosphate-guanosine (CpG) dinucleotides (Surace and Hedrich, 2019). The addition of methyl groups to CpG dinucleotides confers genomic stability and adds another level of tissue-specific transcriptional regulation through the prevention of transcription factor recruitment and the assembly of the transcriptional complex (Hedrich et al., 2017; Surace and Hedrich, 2019).

In 1996, Kim et al. reported the first DNA methylation study in PsA patients (Table 2). Although it included only a relatively small number of patients, it delivered the first evidence suggesting that PBMCs from PsA patients have a specific methylation signature (Kim et al., 1996). Genome-wide DNA methylation of PBMCs was lower in patients with inflammatory arthritis (RA and PsA) off treatment as compared to patients receiving methotrexate or healthy controls and OA patients. This suggests that PsA and RA are associated with DNA hypomethylation that may be reversed by methotrexate treatment. This was later confirmed in RA patients, who exhibited reduced global DNA methylation in T cells and monocytes when comparing with healthy controls. DNA hypomethylation was accompanied by reduced expression of DNA methyltransferase 1 (DNMT1) (De Andres et al., 2015). Similarly, DNA methylation levels are reduced in PBMCs from psoriasis patients (Zhang et al., 2010). In particular, psoriasis patients exhibit DNA hypomethylation of the *IFNG* gene in effector “double negative” T cells that infiltrate the epidermis and contribute to inflammation (Brandt et al., 2017). Recently, several differentially methylated regions (DMRs) were identified and validated by pyrosequencing in sperm cells from PsA patients. Changes in the methylation levels of genes associated with skin/joint disease (*MBP, OSBPL5, SNORD115*, and *HCG26*) and joint disease (*IL22, ELF5, PPP2R2D, PTPRN2*, and *HCG26*) were found (Pollock et al., 2019). These DMRs are mostly associated with cytokine: cytokine receptor interaction and include genes previously associated with PsA, namely the *IL22*, a cytokine produced by IL-23-driven Th17 cells, *PPP2R2* (protein tyrosine phosphatase, receptor type N2) that contains a PsA-associated SNP, and *HCG26* (promoter and body of HLA complex group 26) which is located between the PsA risk loci *MICA* and *MICB* (Stuart et al., 2015; Pollock et al., 2019).

In line with findings in psoriatic disease, abnormal DNA methylation also characterizes other auto-immune/inflammatory disorders, such as systemic lupus erythematosus (SLE), where global DNA hypomethylation of CD4^+^ T cells has been associated with T cell autoreactivity in active lupus patients (Richardson, 1986; Zhang et al., 2013). Also, in SLE patients, DNA hypomethylation CD4^+^ T cell is associated with reduced expression of DNMT1, which correlates with disease activity (Januchowski et al., 2008; Lei et al., 2009). Although data on DNA methylation signatures in PsA are limited, aforementioned preliminary observations are promising and, also in the context of findings from other autoimmune/inflammatory conditions, will trigger further investigation.

### Histone Marks in PsA

In the nucleus, genomic DNA is organized in complex chromatin structures. Chromatin is composed of nucleosomes that contain 2 subunits of histone tetramers formed by histone 2A (H2A), histone 2B (H2B), histone 3 (H3), and histone 4 (H4) that bind to the DNA through electrostatic interactions (Tessarz and Kouzarides, 2014; Prakash and Fournier, 2018). Post-translational modifications to N-terminal amino acid residues within histone proteins, such as, e.g., their acetylation and/or methylation, result in changes to their electric charge and thereby regulate their three-dimensional composition. Through this, histone modifications can mediate transcriptional activation or repression based on the location and nature of post-translational modifications (Hedrich and Tsokos, 2011; Tessarz and Kouzarides, 2014).

Histone acetylation patterns are altered in psoriatic disease (Table 2). PBMCs from PsA patients exhibit decreased H4 (but not H3) acetylation when compared to healthy controls (Ovejero-Benito et al., 2018). Sirtuins are a family of histone deacetylases that contain seven members, namely SIRT1-SIRT7. Their main function is to suppress gene transcription through epigenetic remodeling (Mendes et al., 2017). Sirtuins have been linked with the pathogenesis of PsA. SIRT1, a NAD^+^ dependent Class III histone deacetylase, is involved in the regulation of T cell activation and bone metabolism (Cohen-Kfir et al., 2011; Moon et al., 2013). Anti-Sirt1 autoantibodies were found elevated in the serum of PsA, but not RA patients or healthy controls (Hu et al., 2018). Inhibition of sirtuin-1, using the histone deacetylases (HDAC) inhibitor sirtinol, results in increased H3 and H4 acetylation and reduced secretion of inflammatory chemokines, such as CXCL10, CCL2, and CXCL8. This disrupts inflammatory responses to TNF-α and IL-1β, which are both part of the PsA inflammatory cascades (Orecchia et al., 2011).

The likely involvement of SIRT1 in the molecular pathogenesis of PsA is an example of how epigenetic mechanisms link “trigger events” with inflammation and tissue damage. The modulation of the gastrointestinal environment in mouse models on high-fat diet by oral administration of *Lactobacillus sakei* increases the SIRT1 expression in the liver and colon (Jang et al., 2019). Studies demonstrated that an increase in gut microbiota-derived butyrate is related with increased levels of fibroblast growth factor 21 (FGF21) in the liver, which correlates with the increased expression of SIRT1 in the same tissue (Pant et al., 2017; Kundu et al., 2019). FGF21 plays a role in immune mediated diseases, and its administration reduces the inflammation in collagen-induced arthritis models (Yu et al., 2017). Another example on how diet and microbiota can affect epigenetic events is linked to diets rich in either phytate or fiber, which are metabolized by the microbiota into Inositol-3 phosphate or short chain fatty acids (SCFA), respectively (Wu S.E. et al., 2020). Inositol-3 phosphate acts as inhibitor, while SCFA acts as activating signal for HDAC3 (histone deacetylase 3) in epithelial cells, which contribute to gut-host homeostasis (Wu S.E. et al., 2020). Feeding wild-type conventional and germ-free (GF) mice with phytate they demonstrated the requirement for microbiota in HDAC3 phytate-dependent regulation. A mammalian inositol phosphatase homolog in Bacteroides-derived outer membrane vesicles (OMV) is involved the degradation of dietary phytate and inositol signaling pathway (Stentz et al., 2014). This suggests that commensal microbiota and OMV may play a role in the fine tuning of HDAC3 in gut epithelial cells. Although the effect of HDAC3 has not been studied in PsA specifically, HDAC3 inhibitors were suggested by some authors as potential future drugs for inflammatory diseases, including RA and psoriasis (Angiolilli et al., 2017; Choudhary et al., 2017).

### Non-coding RNAs in PsA

The study of differentially expressed non-coding RNAs, namely micro-RNAs (miRNAs), and their association with the pathogenesis and treatment responses recently moved into the focus of research in PsA (Table 2).

A number of studies reported differential expression of miRNA-146a in PsA patients, and its correlation with clinical response to treatment (Ciccacci et al., 2016; Maharaj et al., 2018; Lin et al., 2019; Wade et al., 2020). Micro-RNA-146a expression is increased in peripheral CD14^+^ monocytes from patients with PsA when compared with psoriasis patients and healthy individuals and correlates with osteoclast activation and bone resorption (Lin et al., 2019). Micro-RNA-146a binds the mRNA 3’-UTR of TNF receptor-associated factor 6 (TRAF6) and the IL-1R-associated kinase (IRAK1), inhibiting their expression (Chatzikyriakidou et al., 2010; Nakasa et al., 2011; Kagiya and Nakamura, 2013). The PAMP and DAMP target TLRs and IL-1 receptors that recruit the myeloid differentiation primary response 88 (MyD88) and IRAK family proteins. Activated IRAK proteins associate with TRAF6 leading to activation of several transcription factors that mediate osteoclast differentiation and inflammation (Jain et al., 2014). Recently, genetic variants affecting miRNA-146a genetic have been associated with PsA (rs2910164 c.60C > G, MIR146A), where the presence of the G allele was found to confer higher susceptibility to Ps and PsA (Caputo et al., 2020a) (Figure 1). Of note, miRNA-146a is associated with other autoimmune diseases namely in RA where the levels of miRNA-146a are increased in circulation and synovial fluid (Bae and Lee, 2018).

Micro-RNA-126-3p and miRNA-941 have been implicated in the pathophysiology of PsA. miRNA-126-3p has been associated with several autoimmune disorders, including RA and PsA (Pelosi et al., 2018; Ormseth et al., 2021). Its expression is downregulated in PsA patients with active disease, and its forced expression induces a decrease in expression of PsA associated RANKL (Pelosi et al., 2018). Micro-RNA-941 is upregulated in CD14^+^ monocytes from PsA patients, and its inhibition abrogates osteoclast differentiation (Pelosi et al., 2018; Lin et al., 2019).

A recent study found increased levels of the TLR7 ligands miRNA-29 and miRNA-Let7b in the synovial fluid of PsA patients. In mice, miRNA-Let7b can trigger skin-to-joint crosstalk. Accordingly, the administration of this miRNA stimulates the expansion of Th1 cells and concomitant production of IFN-γ, which in turn stimulates macrophages (see above). Subsequent glycolytic skin inflammation signals trigger synovial macrophages to produce Th17 polarizing cytokines, which also stimulate RANKL expression and the differentiation of osteoclasts and resulting inflammation and joint erosion (Van Raemdonck et al., 2020).

**FIGURE 2:**
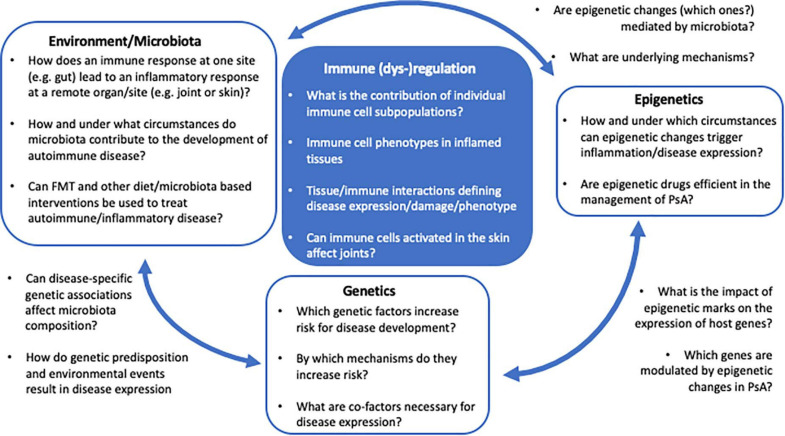
The PsA multifactor model. Several questions remain unanswered regarding the impact of genetics, epigenetics, and environment/microbiota in the immunopathogenesis of PsA and the regulatory mechanism underlying the interaction between these factors.

The current understanding of disease-associated epigenetic marks is limited, and likely only represents the tip of an iceberg. Detailed and in-depth investigation of disease-associated epigenetic signatures in psoriasis and PsA, including various immune and stroma cell types, is required to understand their role in the molecular pathophysiology. Furthermore, based on recent observations, epigenetic patterns may be investigated at a single cell resolution, as clonal expansion of specific inflammatory immune cell population may drive disease and can be missed when addressing “bulk” populations (Figure 2).

## Environmental Triggers and the Microbiome

Environmental factors, such as stress, trauma, infection and/or microbiota/diet are possible triggers of chronic immune activation in genetically predisposed individuals. While some environmentally triggered changes to the epigenome have been mentioned above, the following section will discuss the involvement of gut microbiota in PsA and their contribution as pathogenetic factor in more detail.

Over the past years, increasing attention has been paid to the role of gut microbiota and their effects on immune homeostasis in health and disease. Unfortunately, individual microbiomes are variable and large datasets in various ethnic and cultural groups are necessary to understand what can be considered “normal.” Host-microbiota interactions are complex and very incompletely understood. Still, it is widely accepted that the gut microbiome is involved in the development and maturation of the host’s immune system (reviewed in Parker et al., 2018). After birth, the expression and activation status of pattern recognition receptors in epithelial cells and other cells of mucosal immune system, such as macrophages, NK cells and DCs, keeps changing alongside microbial colonization. Pattern recognition receptors, including toll-like receptors, impact on epithelial cell proliferation and survival, and immune responses (Hooper and Macpherson, 2010; Neal et al., 2012; Parker et al., 2018). Though the exact mechanisms involved are unknown, co-dependency and close interaction between host and gut microbiome are beyond doubt.

The intestinal epithelial barrier has several mechanisms to detect, tolerate and/or limit pathogen invasion. The intestinal environment is composed not only of microbes including bacteria, virus and yeast, but also diet components, microbial metabolites such as short chain fatty acids (SCFA) and trimethylamine N-oxide (TMAO), and bacteria-derived outer membrane vesicles (Omenetti and Pizarro, 2015; Parker et al., 2018). Under physiological circumstances, intestinal flora and host are in homeostasis and both communicate and benefit from this relationship. Dysbiosis refers to a shift in microbiota, resulting in a disturbed intestinal environment in terms of composition and/or abundance of microbes and their products, and subsequent disruption of the communication between microbiota and the host’s immune system (Parker et al., 2018). When dysbiosis occurs, the disturbed intestinal microbiota can stimulate the mucosal immune system, e.g., by recruiting Th17 lymphocytes (Rehaume et al., 2014). Ivanov et al. (2009) reported the significant impact of segmented filamentous bacteria on CD4^+^ helper T cells in the murine intestinal lamina propria, which shifts toward inflammatory Th17 phenotypes. Differentiation of Th17 cells and inhibition of regulatory T cells (Tregs) can induce mucosal inflammation locally, and migration of intestinal T cells can induce inflammation in other organs, including the skin and/or joints (Omenetti and Pizarro, 2015). Indeed, intestinal dysbiosis has been implicated in the pathogenesis of several conditions, such as obesity, IBD, Alzheimer’s disease, and PsA (Scher et al., 2015; Ananthakrishnan et al., 2018; Chen et al., 2020; Toubal et al., 2020). Patients with PsA frequently report gut complications and gut inflammation (Schatteman et al., 1995; Scarpa et al., 2000; Lindqvist et al., 2006; Scher et al., 2015; Ciccia et al., 2016a; Tito et al., 2017) which is reflected by lymphocyte infiltrates in the duodenal mucosa (Lindqvist et al., 2006).

Although larger multi-center studies are required to further investigate and establish microbial patterns associated with PsA, preliminary data point to reduced numbers of gut bacteria, mainly due to reduced *Akkermansia, Ruminococcus*, and *Pseudobutyrivibrio* species in fecal samples of PsA patients when compared with healthy controls (Scher et al., 2015). Tito et al. observed a positive correlation between the abundance of the genus *Dialister* in ileal biopsis from patients with spondylarthritis (SpA) and the Ankylosing Spondylitis Disease Activity Index (ASDAS) (Tito et al., 2017). In psoriasis patients, fecal microbial signatures show an increased abundance of *Phylum firmicutes* (Shapiro et al., 2019).

Several mechanisms have been proposed through which dysbiosis may dysregulate epithelial barriers and trigger autoimmune events. *Bacteroides fragilis* and *Clostridium* spp. promote regulatory T cell responses through their production of short chain fatty acids (SCFA) (Round and Mazmanian, 2010; Atarashi et al., 2011) that activate the G-protein-coupled-receptor GPCR43 (Wu T. et al., 2020). Changes in the PsA gut microbial abundance have been associated with increased levels of IgA in fecal samples, which could result from an exacerbated mucosal immune response (Scher et al., 2015). Furthermore, dysbiosis and associated alterations in intestinal permeability has been reported in PsA (Ciccia et al., 2016a; Nakata et al., 2017; Pianta et al., 2017; Tito et al., 2017). In ankylosing spondylitis, downregulation of the tight junction proteins claudin2 and 4 in the zonula occludens in ileum biopsies has been shown (Ciccia et al., 2015), resulting in increased intestinal permeability and the translocation of bacteria, OMV and related macromolecules to the blood stream. Indeed, serum lipopolysaccharide levels in ankylosing spondylitis patients correlate with tissue inflammation (Ciccia et al., 2015). In PsA, the gut epithelium is characterized by a generally pro-inflammatory environment with expansion of innate lymphoid cells, activation and expansion of Th9 cells and a decrease in the number of Tregs (Ciccia et al., 2016a; Manasson et al., 2020). Aforementioned changes to the gut microbiome can influence the tight balance between effector Th1 and Th17 lymphocytes, which is essential for host defense and involved in PsA pathogenesis (Chudnovskiy et al., 2016; Levy et al., 2017).

Another mechanism through which microbiota can modulate the host’s immune response is miR21-5p expression in intestinal epithelial cells (IECs). Expression of the miRNA-21-5p is induced by commensal bacteria, which modulates intestinal epithelial permeability by targeting the ADP ribosylation factor 4 (ARF4) that regulates tight junction proteins claudin and occludin (Nakata et al., 2017). Expression of miRNA-146a is stimulated by gut bacteria and pro-inflammatory cytokines (namely IL-1β and TNF-α) (Anzola et al., 2018; Du et al., 2018). Thus, in humans and mice, dysbiosis and intestinal inflammation can result in alterations in the expression of miRNA-146a (Runtsch et al., 2015; Mirzakhani et al., 2020), which activates inflammatory pathways in genetically susceptible individuals and may be involved in the propagation of associated events, such as bone alterations. It will be relevant to further explore aforementioned and additional miRNAs as a link between dysbiosis/intestinal inflammation and development of autoimmune/inflammatory disease, including PsA.

Another potential effect of microbiota is linked to the presence of bacterial DNA in the peripheral blood of PsA, AS, and RA patients (Hammad et al., 2019) and synovial fluid from chronic arthritis (Nocton et al., 1994; van der Heijden et al., 1999) and PsA patients (Wang et al., 1999). The presence of DNA in the synovial fluid may be the result of increased permeability of intestinal epithelial barriers. Gut bacteria-derived outer membrane vesicles are a likely carrier of bacterial DNA to the synovium, as they are of markedly smaller size than bacteria which favors their transfer into the blood stream (Stentz et al., 2018; Jones et al., 2020). Outer membrane vesicles are an important player in gut homeostasis, as they interact with the immune system and are able to activate DCs through TLR2. Indeed, TLR2 expression is upregulated in DCs from PsA patients (Candia et al., 2007).

Considering possible effects of dysbiosis on the development of autoimmune/inflammatory disease in genetically susceptible individuals, it is worth noting that alterations to the expression of relevant genes can also have an impact on the microbiota. Polymorphisms in autophagy associated genes, such as *ATG16L1*, have been associated with IBD susceptibility. Impaired autophagy-mediated clearance of intracellular bacteria promotes pro-inflammatory cytokine production, in particular IL-1β, TNF-α and IFN-γ (Lapaquette et al., 2017; Larabi et al., 2020). Interestingly, in PsA, circulating DCs exhibit increased levels of ATG16L1 and LL37, both molecules involved in immune responses against intracellular bacteria (Wenink et al., 2011), further suggesting that a deregulated response to bacteria and possibly autophagy pathways may be involved in the pathogenesis. Lin et al. demonstrated that *Lewis* rats expressing human HLA-B27 and hβ2m have altered intestinal microbiota as compared to wild type animals. A further extensive metabolomic analysis of rat cecal content showed reduced levels of short chain fatty acids (SCFA) (Lin et al., 2014). Treatment with the SCFA propionate attenuates the development of HLA-associated inflammatory disease (Asquith et al., 2017). Furthermore, in rats, HLA-B27 is associated with reduced expression of the free fatty acid receptor FFAR3, through which microbiota products, mainly short chain fatty acids, stimulate inflammatory responses (Ang et al., 2016; Asquith et al., 2017). The stimulation of FFAR3 in PsA synovial fibroblasts mediates increased expression of IL-6 when compared to healthy controls (Frommer et al., 2015).

The potential clinical implications of altered gut microbiota in PsA are underscored by a report of a female patient with severe and disease-modifying antirheumatic drugs (DMARD)-dependent PsA, who reached sustained remission off medication after receiving fecal-microbial transplantation (FMT) for symptomatic *Clostrodium difficile* infection (Selvanderan et al., 2019). Although this is a single case and could be the result of a coincidence, it is not a surprising outcome given the reports associating dysbiosis and PsA. Currently, a randomized double-blinded placebo-controlled trial testing FMT in PsA patients with active peripheral disease requiring treatment with the DMARD methotrexate is under way (Kragsnaes et al., 2018). The study is investigating the composition of gut microbiota, DAI and intestinal permeability (lactulose/mannitol test) before and after FMT (Kragsnaes et al., 2018).

The relationship between commensal bacteria and the host’s immune system is complex and not well understood. While it appears certain that the microbiome plays a critical role in the development of autoimmune disease, key questions remain unanswered regarding the direct and indirect (through epigenetic changes?) impact of dysbiosis on the development of inflammatory disease, including PsA (Figure 2). Deciphering environmental triggers and associated mechanisms promoting autoimmune disease in genetically predisposed individuals will allow for the introduction of new therapeutic or even disease preventive interventions, potentially including alterations to the microbiota (e.g., through FMT?).

## Altered Synovial Vascularization in PsA

**FIGURE 3:**
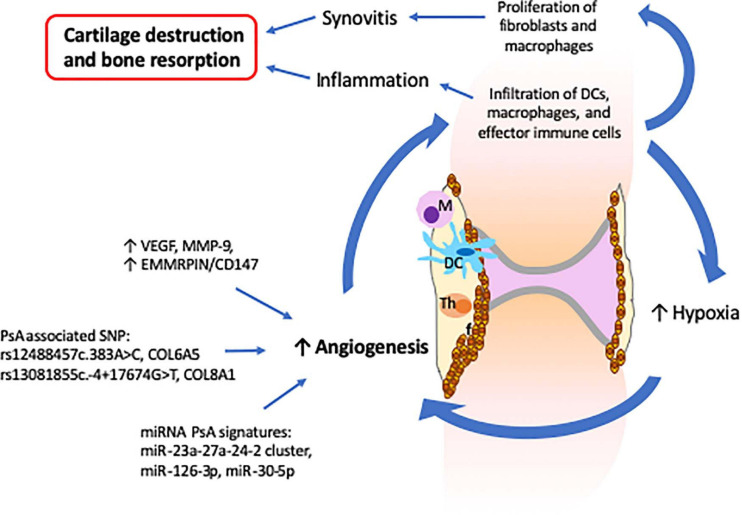
Altered synovial vascularization in psoriatic arthritis is critical for the development of synovitis and inflammation which result in cartilage destruction and bone resorption. The PsA synovium is characterized by an increased vascularization that facilitates the infiltration of innate and effector immune cells. The increase of immune cells together with the proliferation of fibroblasts leads to a hypoxia environment that further stimulates angiogenesis and infiltration of more immune cells, inducing propagation of inflammation. The increased levels of VEGF, MMP-9, and EMMRPIN/CD147 observed in PsA synovium together with the several SNP and miRNA signatures associated with angiogenesis support the critical role of angiogenesis in the development of psoriatic arthritis.

In a healthy joint, the synovial membrane produces synovial fluid that lubricates the joint during movement and nourishes avascular cartilage (Mathiessen and Conaghan, 2017). The synovial membrane consists mainly of a layer of synovial fibroblasts, synovial macrophages and a second layer of connective tissues that provides support to a network of nerves and blood vessels that provide oxygen, nutrients and immune-drainage (Mathiessen and Conaghan, 2017). In PsA, the synovial composition is altered and characterized by increased vascularization, proliferation of fibroblasts, and infiltration of innate (DCs and macrophages) and activated adaptive (T and B cells) immune cells (Cafaro and Mcinnes, 2018). In this context, the prominent vascularization is an important feature of PsA as it facilitates the infiltration of immune cells that contribute to the propagation of the inflammatory process (Zhang et al., 2012) (Figure 3). Furthermore, increased infiltration of immune cells induces a hypoxic micro-environment, which in turn promotes angiogenesis, resulting in a self-promoting circle (Del Rey et al., 2009). Lastly, synovial tissue and infiltrating immune cells (increased in PsA when compared with RA) express vascular endothelial growth factor (VEGF) and MMP-9 proteins that are involved in the proliferation of capillaries and EMMRPIN/CD147, which positively regulates the expression of VEGF and MMPs (Yurchenko et al., 2010; Yamamoto, 2013; Rahat et al., 2020) (Figure 3).

Recently, links between synovial pathology and genetic and epigenetic factors have been established. Psoriasis and PsA associated polymorphisms in collagen genes, namely (rs12488457c.383A > C, *COL6A5*) and (rs13081855c.-4 + 17674G > T, *COL8A1*), are associated with increased angiogenesis and vascular proliferation (Caputo et al., 2020a). Plasma extracellular vesicles isolated from PsA patients exhibit reduced levels of miR-30-5p as compared with psoriatic skin (Pasquali et al., 2020). As miRNA-30-5p is involved in the inhibition of angiogenesis, decreased expression in PsA may contribute to increased synovial vascularization that distinguishes psoriasis from PsA. Along these lines, miRNA-126-3p and miRNA-23a-27a-24-2, which are involved in angiogenesis, were also found to be differently expressed in PsA as compared to RA and osteoarthritis patients (Pelosi et al., 2018; Wade et al., 2019b).

## Patient Stratification, Current and Future Treatments

Reflecting the complex pathophysiology of psoriasis and PsA, treatment can be challenging and trial-and-error approaches have to be taken in the absence of tools for molecular stratification of patients. Psoriatic arthritis patients can develop arthritis before the onset of skin symptoms, potentially resulting in initially incorrect classification as other forms of chronic arthritis. As classical DMARDs are less efficient in PsA when compared to, e.g., RA, this can affect patients’ quality of life and contribute to damage accrual (Lubrano et al., 2018). Thus, aforementioned steps toward a more complete understanding of the molecular pathophysiology and inter-individual differences are of key importance and will, while additional studies are urgently needed, contribute to future patient stratification and new, individualized and target-directed treatments for psoriasis and PsA.

Currently available target-directed treatments are limited. Biopharmaceutical treatments available for PsA and psoriasis target aberrant inflammatory responses, namely excess of pro-inflammatory cytokine production (Sakkas et al., 2019). Approved therapeutics for PsA include recombinant monoclonal antibodies targeting the p40 subunit of IL-12 and IL-23 (ustekinumab), IL-17A (ixekizumab and secukinumab), and TNF-α (adalimumab, certolizumab, golimumab, infliximab), and the fusion protein etanercept consisting in the fusion protein of two extracellular ligand-binding portions of the human TNF receptor linked to the Fc domain of an IgG antibody (Sakkas et al., 2019).

Recently, because of abovementioned pathological activation of Janus kinases (JAKs) in PsA, small molecule JAK inhibitors have been tested and recently approved by the U.S. Food and Drug Administration (FDA) and European Medicines Agency (EMA) (Sakkas et al., 2019). Janus kinases are a family of proteins with tyrosine kinase activity that includes JAK1, JAK2, JAK3, and TYK2 that become activated in response to type I and type II cytokine recruitment to their cell-surface receptors (El Jammal et al., 2020). As a result, JAKs phosphorylate tyrosine residues of JAKs and/or adjacent molecules, including STAT family transcription factors that regulate the expression of, e.g., cytokine genes (Hodge et al., 2016). The currently available JAK inhibitor used for the treatment of PsA, tofacitinib, preferentially acts on JAK1 and JAK3 (Scott, 2013), impairing signaling cascades mainly triggered by cytokines IL-6, IL-7, IL-10, IL-15, and IL-21, as well as IFN-α and IFN-γ (Hodge et al., 2016).

While based on preliminary observations, epigenetic alterations observed in individual patients with PsA may be used for future patient stratification. DNA methylation patterns associated with variable drug responses and disease progression in PsA, suggesting their use as molecular biomarker for patient stratification and individualized treatment (Kim et al., 1996; Pollock et al., 2019). Variable methylation of lysin 4 at histone H3 (H3K4) discriminates between responders and non-responders to biopharmaceutical treatments (namely adalimumab, ixekizumab, secukinumab and ustekinumab) at 3 and 6 months (Ovejero-Benito et al., 2018).

As epigenetic alterations associate with PsA and likely contribute to altered gene expression, their correction may offer new therapeutic avenues in PsA and other autoimmune/inflammatory conditions. Indeed, effects of pharmacological inhibitors on epigenetic modifier enzymes have been explored in PsA. The histone deacetylase (HDAC) inhibitors trichostatin A (TSA) and JQ1 (a novel panBET bromodomain HAT inhibitor) reduce the transformation of Tregs into pro-inflammatory Th17 cells, subsequently reducing IL-17A secretion (Bovenschen et al., 2011; Hammitzsch et al., 2015). The inhibition of bromodomains of CBP (CREB-binding protein)/p300 with the small molecule bromodomain inhibitor CBP30, results in reduced IL-17A secretion in PsA patients (Hammitzsch et al., 2015).

Indeed, even currently available and frequently used therapeutics affect the epigenetic code. The use of the classical DMARD methotrexate reverses DNA hypomethylation in PBMCs from PsA patients (Kim et al., 1996). However, based on clinical experience, classical DMARDs are of limited use in PsA and inferior to, e.g., aforementioned cytokine blocking strategies.

Lastly, preliminary evidence suggests microbiota as environmental modulators of epigenetic marks and (resulting) immune responses. Thus, alterations to the microbiome may limit inflammation in psoriasis and PsA patients, or even delay or prevent disease onset in genetically predisposed individuals, promising significant potential to improve disease outcomes and population health. Indeed, first reports promise potential for FMT or eradication of pathogenic microbiota in patients with autoimmune/inflammatory conditions (Kragsnaes et al., 2018; Selvanderan et al., 2019). However, currently, we are only beginning to understand the “normal” composition of microbiota and effects of dysbiosis.

The discovery and introduction of biopharmaceutical drugs currently used in PsA progressed hand-in-hand with our pathophysiological understanding of PsA. Treatment response is determined by an individual’s genetic profile, additional host factors, including the microbiota, and the environment (Sayers et al., 2018; Scher et al., 2020; Morelli et al., 2021). Understanding the complex interplay between these (and additional) factors will deliver new treatment targets. In parallel, clinical and molecular assessment of currently used and new drugs will likely reveal the involvement of molecular pathways in PsA.

## Conclusion

Psoriatic arthritis is a mixed pattern condition, characterized by an interplay between innate and adaptive immune mechanisms resulting in the development and propagation of inflammation. Although IL-23/Th17 axis has been identified and has a critical target for PsA management, the exact pathophysiological mechanisms underlying disease expression remain incompletely understood and need further investigation. Genetic variations associated with the development of psoriasis and PsA have been described, but monogenic disease is rare and mostly due to mutations in *CARD14*. Most associated genetic variants, however, contribute to increased susceptibility, while individually not being strong enough to confer disease. Environmental factors, including changes to microbiota, may alter epigenetic marks, gene expression profiles, and immune responses in genetically predisposed individuals. Currently available multifactorial disease models are incomplete and likely oversimplified (Figure 2). To allow introduction of disease preventive and/or new target-directed interventions, a better/complete understanding of molecular disease mechanisms including genetic susceptibility, environmental triggers, and resulting pathological immuneactivation is necessary. The study of microbiota and epigenetic modifications in the context of genetic factors will open new avenues for the development of disease- and outcome-specific biomarkers, preventative interventions, and new target-directed individualized therapies. These will allow for the induction of early remission and decrease an individual patient’s economic burden for the healthcare system.

## Author Contributions

AC and CH wrote and edited the review.

## Conflict of Interest

The authors declare that the research was conducted in the absence of any commercial or financial relationships that could be construed as a potential conflict of interest.
